# A retrospective review of 61 cases of adenomatoid odontogenic tumour seen in five tertiary health facilities in Nigeria

**DOI:** 10.11604/pamj.2016.24.102.9400

**Published:** 2016-05-31

**Authors:** Akinyele Olumuyiwa Adisa, Ahmed Oluwatoyin Lawal, Olajumoke Ajibola Effiom, Olujide Oladele Soyele, Olufemi Gbenga Omitola, Adetokunbo Olawuyi, Benjamin Fomete

**Affiliations:** 1 Department of Oral Pathology, College of Medicine, University of Ibadan, Ibadan, Nigeria; 2 Department of Oral and Maxillofacial Pathology & Biology, College of Medicine, University of Lagos, Lagos, Nigeria; 3 Department of Oral Maxillo-facial Surgery and Oral Pathology, Obafemi Awolowo University, Ile-Ife, Nigeria; 4 Department of Oral Pathology and Biology, University of Port Harcourt, Port Harcourt, Nigeria; 5 Maxillofacial Surgery Departments, Ahmadu Bello University Teaching Hospital, Zaria, Nigeria

**Keywords:** Adenomatoid odontogenic tumour, radiology, impacted teeth

## Abstract

**Introduction:**

Adenomatoid odontogenic tumor (AOT) is a benign lesion originating from the dental lamina or its remnants. It is a relatively uncommon neoplasm representing about 3% of all odontogenic tumors. The aim of this study was to examine the clinical and radiological characteristics of AOTs in five major tertiary centres in Nigeria.

**Methods:**

Archival hospital-based data stores of five tertiary health facilities in Nigeria were accessed. Case files and biopsy records were retrieved to obtain relevant information. Data was collected according to a proforma for standardization and entered into and analysed using SPSS for Windows (version 20.0; SPSS Inc. Chicago, IL).

**Results:**

61 (4.5%) cases of AOT were documented. The age range was 8-46 years with a mean age of 20.4±9.9 years. Male: Female ratio was 1:1.3. The anterior maxilla had 34 (55.8%) cases and the anterior mandible had 20 (32.8%) cases. 40 (65.6%) follicular cases, 20 (32.8%) extra-follicular cases and 1(1.6%) extra-osseous case were found. 31 cases (61.1%) were associated with impacted teeth and the upper canine was involved in 19 (57.6%) cases.

**Conclusion:**

This study showed AOT to be more common in the maxilla, more in females, most often associated with impacted canines, however, the suggestion of AOT being a “Two third tumour” was not observed in this study.

## Introduction

Adenomatoid odontogenic tumor (AOT) is a benign lesion originating from the dental lamina or its remnants [1]. It is a relatively uncommon neoplasm representing about 3% of all odontogenic tumors and was first described by Steensland in 1905 [2, 3]. AOT was previously described as; adenoameloblastoma, ameloblastic adenomatoid tumor, adamantinoma, epithelioma adamantinum or teratomatous odontoma [4]. However, it was Philipsen and Birn who first introduced the term “AOT” in 1969 and this was later adopted by WHO in her first histological typing of odontogenic tumors, jaw cysts and allied lesions in 1971 [1, 5]. AOT has been reported to constitute about 1.2% of odontogenic tumor's (OTs) in Caucasians and up to 9% of OTs in Black Africans [6, 7]. The tumor is most often diagnosed in the second decade of life and women are about twice as often affected than men [6]. The tumor is sometimes described as the “two third tumour”, since, two third of cases occur in the maxilla, two third affects young females, two third is associated with an un-erupted tooth and two third of affected teeth are canines [8, 9]. AOT occurs in three distinct clinico-topographic variants: follicular, extrafollicular and peripheral. Philipsen and Reichart had in a previous study, reported that the follicular and extrafollicular variants (both of which are intra-osseous), accounted for about 96% of all AOTs seen and of these, 71% were follicular [10]. Presumably, because of its relative rarity, previous reports on AOT were either case reports or small case series [1, 5, 6]. Moreso, only few studies on the demography of AOT can be found in African literature, though several studies had previously reported AOT as part of larger series on odontogenic tumours [11–13]. The aim of this study was to examine the clinical and radiological characteristics of AOTs in five major tertiary centres in Nigeria.

## Methods

Archival hospital-based data stores of Tertiary Health facilities were accessed via collaborative contacts from Lagos University Teaching Hospital Lagos, University College Hospital Ibadan, Obafemi Awolowo University Teaching Hospital Ife, Ahmadu Bello University Zaria Kaduna state and University of Port Harcourt Teaching Hospital Rivers state. Case files and biopsy records were retrieved to obtain age, gender, site, clinical symptoms and radiographic appearances. Data was collected according to a proforma for standardization and entered into and analysed using SPSS for Windows (version 20.0; SPSS Inc. Chicago, IL).

## Results

Sixty-one cases of AOT were documented from this study out of a total of 1369 OTs from the 5 centres representing 4.5% of all OTs reported. The age range of included patients was 8-46 years with the mean age being 20.4±9.9 years and peak age incidence in the second decade (Table 1). There were 26 (42.6%) males and 35 (57.4%) females (male: female ratio 1:1.3). The mean duration of tumor evolution before presentation and treatment was 19.1±18.6 months. The site of predilection was the anterior maxilla, with 34 (55.8%) cases, followed by the anterior mandible with 20 (32.8%) cases. The posterior mandible presented with 7 (11.5%) but the posterior maxilla was not involved in any of the cases we documented. We found 40 (65.6%) follicular AOTs, 20 (32.8%) extra-follicular cases and only 1(1.6%) extra-osseous case of AOT. Thirty-three cases (61.1% of the 54 cases with available information on impacted teeth) were associated with impacted teeth and the upper canine was involved in 19 cases representing 57.6% of all impacted teeth. The most common radiographic presentation was that of a well-delineated unilocular radiolucency with a single opacity in 57.4% of patients, while 9.8% had a unilocular radiolucency with multiple opacities within them and 3.3% had multilocular radiolucency with no opacities in them at all. Table 2 shows distribution according to the clinic-topographic variants.

**Table 1:** Age group distribution of all AOT cases

Age group	Frequencies (%)
0-9	4.0 (6.6)
10-19	36.0 (59.0)
20-29	9.0 (14.8)
30-39	6.09 (.8)
40-49	5.0 (8.2)
Total	60.0 (98.4)
Missing	1.0 (1.6)
Overall total	61(100.0)

**Table 2:** Distribution according to the clinic-topographic variant of AOT

	No (%)	Mean age (± SD)	Peak age	M: F (%)	Max: mand (%)	Impacted teeth N (%)	% of impacted Upper anterior
Foll.	40 (65.6)	18.2 (± 8.2)	10-19	M,17 (42.5): F, 13 (57.5)	Max, 26 (65): Man, 14 (45)	40 (100.0)	25 (65.0)
Extra-foll.	20 (32.8)	24.7 (± 11.9)	10-19	M,8 (40): F, 12 (60)	Max, 7 (35): Man, 13 (65)	Nil	Nil
ST	1 (1.6)	18.0	10-19	male		Nil	Nil

M=male, F=female, Max=maxilla, man=mandible, Foll=follicular, Extra-foll=extra-follicular, ST=soft tissue

## Discussion

The relative rarity of AOT previously reported by other authors was corroborated by this study that found that AOT represented 4.5% of all OTs. Similar studies from Mexico [14], China [15] and California [16] found the relative occurrence of AOT amongst total OTs, as 7.1%, 2.1% and 1.7% respectively. The female predilection reported in previous studies was also observed in this study, however, the female: male ratio of 1.3: 1 obtained in this study did not reflect the marked female preponderance previously reported especially in Asia. Toida et al [17] in Japan and Mendis [18] in Sri-Lanka had previously reported female: male ratio of 3.0: 1 and 3.2: 1. On the contrary, Arotiba et al [19] in a previous study from Nigeria and Becker et al [20] in a review of 267 previous and 5 new cases both obtained female: male ratio of 1.4: 1 which were more in line with our finding. AOT is a lesion that is most often diagnosed in young patients especially those in the second decade of life and it is said to be rare in patients above 30 years of age [21]. The mean age of 20.4 years from this study was higher than most other reports. De Matos et al [21] in a retrospective review of 15 cases from Brazil got a lower mean age of 16.2 years, while the study by Becker et al [20] obtained a mean age of 18.4 years. Mean ages from studies by Buchner et al [16] from Califonia, and Lu et al [22] from China were 20.2 years and 22.6 years respectively, which were more in keeping with this study. Also, only 18% of AOTs were found in patients above 30 years of age, which was in agreement with previous reports that AOT is rare above 30 years [19–21]. AOT occurs predominantly in the maxilla and anterior jaws are much more affected than the posterior jaws [6]. Although, some reports from Nigeria [19, 23] suggested a mandibular predilection for AOT, more recent studies from Nigeria by Arotiba et al [19] and Effiom et al [24] agreed with an anterior maxillary preponderance. However it must be noted that the 44.3% mandibular presentation seen in this study is one of the highest reported in literature. Therefore, the possibility that Nigerian AOTs have a higher mandibular occurrence cannot be ruled out and the possible reason(s) why Nigerian AOTs seem to have a higher mandibular presentation than seen in most other parts of the world needs further investigation. The higher percentage of extra-follicular variant of AOT (32.8%) obtained in this study may partly explain this finding; Arotiba et al [19] had previously reported a mandibular predilection in extra-follicular variant (mandible: maxilla = 2.3:1.) which was also observed in this study (mandible: maxilla = 1.9:1). The follicular clinico-topographic variant of AOT that has been widely reported to be the most common variant of AOT was also the most common in this study with 65.6% of the cases. However, the finding of 32.8% extra-follicular AOT was higher than those reported by most other studies; Becker et al [20] and Philipsen [25] had reported 27% and 26.9% extra-follicular AOT occurrence respectively. Sixty-one percent of our cases were associated with impacted teeth out of which over 70% were canines. This finding was in keeping with previous studies which reported that AOTs were associated with impacted canines and that the most commonly impacted teeth were the maxillary canine [19–21].

## Conclusion

This study generally observed the clinical trend of AOT being more common in the maxilla, more prevalent in females, most often associated with impacted canines and presenting mostly as the follicular clinico-topographic variant. However, the suggestion that AOT is a “Two third tumour” was not found in this study. We suggest that more extra-follicular AOTs seen in this study could account for a relative higher percentage of mandibular presentation and a relatively higher mean age compared with previous studies. Further studies are suggested to ascertain the reason(s) why Nigerian AOT cases have relatively higher mandibular occurrence compared to other climes.

### What is known about this topic

- AOT is a relative rare tumour representing about 3% of odontogenic tumours;
- AOT affects women almost twice as often as it affects men;
- AOT occurs most often in the second decade of life

### What this study adds

- This study showed that there might be slight differences in demography of AOT from region to region;
- The extra-follicular AOT is relatively more common in Nigerian patients compared with those from other climes;
- The “Two third tumour” notion generally ascribed to AOT may not be applicable to Nigerian cases.
